# The NKI-Rockland Sample: A Model for Accelerating the Pace of Discovery Science in Psychiatry

**DOI:** 10.3389/fnins.2012.00152

**Published:** 2012-10-16

**Authors:** Kate Brody Nooner, Stanley J. Colcombe, Russell H. Tobe, Maarten Mennes, Melissa M. Benedict, Alexis L. Moreno, Laura J. Panek, Shaquanna Brown, Stephen T. Zavitz, Qingyang Li, Sharad Sikka, David Gutman, Saroja Bangaru, Rochelle Tziona Schlachter, Stephanie M. Kamiel, Ayesha R. Anwar, Caitlin M. Hinz, Michelle S. Kaplan, Anna B. Rachlin, Samantha Adelsberg, Brian Cheung, Ranjit Khanuja, Chaogan Yan, Cameron C. Craddock, Vincent Calhoun, William Courtney, Margaret King, Dylan Wood, Christine L. Cox, A. M. Clare Kelly, Adriana Di Martino, Eva Petkova, Philip T. Reiss, Nancy Duan, Dawn Thomsen, Bharat Biswal, Barbara Coffey, Matthew J. Hoptman, Daniel C. Javitt, Nunzio Pomara, John J. Sidtis, Harold S. Koplewicz, Francisco Xavier Castellanos, Bennett L. Leventhal, Michael P. Milham

**Affiliations:** ^1^Nathan S. Kline Institute for Psychiatric ResearchOrangeburg, NY, USA; ^2^Psychology Department, University of North CarolinaWilmington, NC, USA; ^3^Phyllis Green and Randolph Cowen Institute for Pediatric Neuroscience, New York University Child Study Center, New York University Langone Medical CenterNew York, NY, USA; ^4^Donders Institute for Brain, Cognition and Behavior, Department of Cognitive Neuroscience, Radboud University Nijmegen Medical CenterNijmegen, Netherlands; ^5^Center for the Developing Brain, Child Mind InstituteNew York, NY, USA; ^6^Department of Psychiatry, New York University Langone Medical CenterNew York, NY, USA; ^7^Virginia Tech Carilion Research InstituteRoanoke, VA, USA; ^8^Department of Psychiatry, University of New MexicoAlbuquerque, NM, USA; ^9^The Mind Research NetworkAlbuquerque, NM, USA; ^10^Department of Radiology, New Jersey Medical SchoolNewark, NJ, USA; ^11^Department of Psychiatry, Columbia University College of Physicians and SurgeonsNew York, NY, USA

**Keywords:** fMRI, DTI, lifespan, brain, phenotype, psychiatry, discovery, open science

## Abstract

The National Institute of Mental Health strategic plan for advancing psychiatric neuroscience calls for an acceleration of discovery and the delineation of developmental trajectories for risk and resilience across the lifespan. To attain these objectives, sufficiently powered datasets with broad and deep phenotypic characterization, state-of-the-art neuroimaging, and genetic samples must be generated and made openly available to the scientific community. The enhanced Nathan Kline Institute-Rockland Sample (NKI-RS) is a response to this need. NKI-RS is an ongoing, institutionally centered endeavor aimed at creating a large-scale (*N* > 1000), deeply phenotyped, community-ascertained, lifespan sample (ages 6–85 years old) with advanced neuroimaging and genetics. These data will be publically shared, openly, and prospectively (i.e., on a weekly basis). Herein, we describe the conceptual basis of the NKI-RS, including study design, sampling considerations, and steps to synchronize phenotypic and neuroimaging assessment. Additionally, we describe our process for sharing the data with the scientific community while protecting participant confidentiality, maintaining an adequate database, and certifying data integrity. The pilot phase of the NKI-RS, including challenges in recruiting, characterizing, imaging, and sharing data, is discussed while also explaining how this experience informed the final design of the enhanced NKI-RS. It is our hope that familiarity with the conceptual underpinnings of the enhanced NKI-RS will facilitate harmonization with future data collection efforts aimed at advancing psychiatric neuroscience and nosology.

## Introduction

Discovery science promises to transform our understanding of human brain function and the impact of neuropsychiatric illness. Traditionally focused on the generation and testing of specific hypotheses, the neuroimaging community is increasingly realizing the value of data exploration techniques capable of uncovering previously unappreciated links between behavior and brain function (Van Horn and Gazzaniga, 2002; Bilder et al., 2009; Cichon et al., 2009; Biswal et al., 2010; Van Dijk et al., 2010). Beyond the identification of novel brain-behavior associations, discovery approaches have the potential to provide the bases for normative trajectories of brain structure and function across the lifespan (Giedd, 1999, 2004; Gogtay et al., 2004; Sowell et al., 2004; Dosenbach et al., 2010; Giedd and Rapoport, 2010; Thompson et al., 2011). Similar to the use of normative physical growth (weight, height) charts in pediatric medicine (Falkner, 1958; Nellhaus, 1968), such trajectories would facilitate the identification and characterization of pathophysiological processes contributing to the emergence of neuropsychiatric illness (Castellanos et al., 2002; Ge et al., 2002; Evans and Brain Development Cooperative Group, 2006; Shaw et al., 2006a,b, 2008, 2010; Gogtay et al., 2007; Dosenbach et al., 2010; Giedd and Rapoport, 2010; Giedd et al., 2010; Thompson et al., 2011). Potentially most exciting is the possibility of revealing markers in early life that have predictive value for the later emergence of illness (Riverol and López, 2011; Shim and Morris, 2011; Taber et al., 2011). Whether during childhood, early adulthood (e.g., Autism, Schizophrenia), or later in life (e.g., Alzheimer’s Disease, Parkinson’s Disease), identification of early biomarkers could transform the delivery of health care by helping to tailor resources and technology to the needs of an individual (i.e., personalized medicine), thus maximizing the likelihood of success for prevention and early intervention strategies.

Successful implementation of discovery science in the imaging community hinges on the accrual of large-scale imaging datasets from individuals who are phenotyped both deeply and broadly (Gogtay et al., 2004; Tracy, 2008; Lanktree et al., 2010). Unfortunately, such datasets are rare. An obvious initial hurdle is the cost of neuroimaging. Magnetic Resonance Imaging (MRI) scanning for research costs between $400 and $750 per hour throughout the world. Unlike genetics, where good quality samples can be obtained nearly anywhere with relative ease, the acquisition of high quality MRI data can be impacted by a variety of issues ranging from scanner-related variation to human factors. Data loss due to factors such as motion is substantial, particularly in child, aging, and clinical populations (Epstein et al., 2007; Power et al., 2012; Van Dijk et al., 2012). Scanner-related anxiety (e.g., claustrophobia) and difficulties following instructions (e.g., participants with severe autism, intellectual disability, psychosis, depression, or mania) represent additional obstacles to obtaining high quality data. Accordingly, funding for sufficiently large samples is beyond the scale of most grant mechanisms. Additionally, there are numerous impediments to deep and broad phenotypic characterizations of large groups of individuals, including recruitment and assessment costs, participant burden, staffing requirements (especially for handling developing and aging populations), and data management (e.g., collection, scoring, storage, retrieval).

The comprehensive characterization of human brain function and structure across the lifespan carries additional challenges with respect to experimental design (National Institute of Mental Health, 2008; Giedd and Rapoport, 2010). Imaging studies tend to focus on narrow comparisons (e.g., pediatrics vs. young adult, or adult vs. aging), precluding there presentation of the full spectrum of typical development, maturation, and aging within a single study (Sowell et al., 2004; Evans and Brain Development Cooperative Group, 2006; Fair et al., 2007; Dosenbach et al., 2010; Tamnes et al., 2010; Allen et al., 2011). This limitation is likely due to the logistical and financial challenges associated with the large sample sizes required to adequately encompass the lifespan with sufficient coverage to achieve statistically significant results. Moreover, the scarcity of imaging and phenotypic assessment tools validated for use across the entire lifespan hampers effective and meaningful acquisition of such data. Additionally, most researchers tend to gravitate toward the beginning or the end of the lifespan, where age-related changes are greatest (Thompson et al., 2011). This produces gaps in our understanding of age-related trajectories of brain structure and function across the lifespan.

The Nathan S. Kline Institute for Psychiatric Research (NKI), funded and operated by the New York State Office of Mental Health, is attempting to address the challenges and capitalize on the opportunities of a lifespan study through the creation of the Nathan Kline Institute-Rockland Sample (NKI-RS). The NKI-RS is intended to be a large-scale, community-ascertained lifespan sample comprised of neuroimaging and genetic data coupled with neurocognitive, physiologic, behavioral, and psychiatric measurements. This initiative brings together researchers from a broad range of disciplines (e.g., basic and systems neuroscience, biostatistics, engineering, computer science, psychiatry, psychology, social work), with interests spanning a range of disorders. The NKI-RS group is focused on developing a unique neuroimaging and genetic sample, linked with descriptive metadata that incorporates solutions for the many challenges facing discovery. First, the age range for the design spans human development from childhood to late adulthood (6–85 years old). Second, the project is applying state-of-the-art resting-state structural and functional MRI (R-fMRI) and diffusion tensor imaging (DTI) techniques (Feinberg et al., 2010; Moeller et al., 2010; Feinberg and Yacoub, 2012; Smith et al., 2012), which minimize the obsolescence of these data along with participant burden. Third, imaging and genetic data are accompanied by a comprehensive phenotypic characterization (e.g., psychiatric, neurocognitive, psychological, and behavioral), to facilitate identification of developmental patterns (Evans and Brain Development Cooperative Group, 2006; Shaw et al., 2006a,b; Giedd and Rapoport, 2010; Giedd et al., 2010). Fourth, the project is grounded on the principles of open neuroscience, with the goal of prospective, pre-publication sharing of all collected data. Finally, while it is generally common practice in imaging studies to overlook concerns about the representativeness of datasets (Szklo, 1998; Evans and Brain Development Cooperative Group, 2006), the second phase of the NKI-RS (i.e., the enhanced NKI-RS) has been designed as a community-ascertained sample closely paralleling U.S. demographic distributions, thus, minimizing potential sampling biases and maximizing representativeness.

While no single effort can create the large-scale datasets necessary to deliver the entirety of normative assessments of the lifespan, it is our intent that this initial effort will highlight the challenges and provide a model through which such data can be acquired and shared. In the following sections, we elaborate on the challenges presented by discovery science and describe the strategies we have adopted in response. We discuss design considerations related to sampling, assessment, and other methodological choices that best characterize brain structure and function across the lifespan and which are most amenable to data sharing. Last, we discuss the specific steps we took to create the first, pilot phase of the NKI-RS, and then describe the conceptual underpinnings of phase two, the enhanced NKI-RS, now being executed.

## Motivation and Challenges for a Lifespan Sample Design

The developmental origins of most neuropsychiatric illnesses are increasingly being appreciated. Nearly 75% of mental illness in adults originates prior to age 24 years (Kessler et al., 2005), and numerous links have been identified between the presence of childhood psychiatric problems and the later onset of adult illness (e.g., pediatric anxiety is associated with increased risk of adult depression; Drevets, 2003; Milham et al., 2005). Whether considering disorders affecting children, adolescents, adults, or the elderly, early detection of disease risk and/or onset is the critical first step in prevention and treatment, respectively (Kessler et al., 2005; Kessler and Wang, 2008). In this regard, the imaging community is increasingly hopeful that normative assessments of brain development, maturation, and aging can be obtained (Evans and Brain Development Cooperative Group, 2006; Fair et al., 2007, 2008, 2009; Church et al., 2009a,b; Kelly et al., 2009; Dosenbach et al., 2010; Giedd and Rapoport, 2010; Zuo et al., 2010; Allen et al., 2011). We anticipate that these normative trajectories will facilitate the identification of markers of pathologic development capable of 1 day informing multiple aspects of clinical assessment and decision-making – ranging from determinations of risk, diagnosis, and prognosis, to the selection and timing of interventions, as well as treatment response monitoring.

While conceptually attractive, building a large-scale imaging dataset that comprehensively samples the lifespan poses daunting challenges. The gold standard for studies of trajectories is the longitudinal design (Kraemer et al., 2000; Thompson et al., 2011). Unfortunately such designs are expensive and generally impractical due to time requirements and cumulative attrition (e.g., loss to follow-up from one time-point to the next, missing data).

A more tractable approach is a lifespan, cross-sectional design that assesses individuals spanning a broad age range to infer developmental, maturational, and aging trajectories. Although more practical, cross-sectional methods can be biased by differential recruitment along the lifespan (e.g., unintended differences in socioeconomic, intellectual, or behavioral characteristics among age-cohorts; Kraemer et al., 2000; Pediatric Imaging, Neurocognition, and Genetics (PING), 2011; Thompson et al., 2011). Hybrid, longitudinal, cross-lag designs that involve sampling individuals cross-sectionally across the lifespan, but following each of them longitudinally, albeit for briefer periods (e.g., 3 or 5 years), hold the greatest potential (Shaw et al., 2010; Thompson et al., 2011). However, these designs still engender significant costs and are hindered by increased potential for data loss.

## From Labs to Collaboratives

It is fair to say that the majority of advances in clinical neuroscience over the past century have emerged through the accumulated contributions of individual labs, each collecting, analyzing, and interpreting its own data independently. However, as the scale and complexity of scientific inquiry increase, collaborative efforts are increasingly essential in order to attain samples of sufficient size and adequate statistical power.

A number of models have emerged to foster the necessary collaboration. For example, the multi-investigator, multi-center model, commonly employed by the pharmaceutical industry, has been effectively implemented by efforts such as the Biomedical Informatics Research Network (BIRN; Helmer et al., 2011) or the Alzheimer Disease Neuroimaging Initiative (ADNI; Mueller et al., 2005). Although successful, such initiatives typically require considerable investment, limiting their growth. Recently, The Brain Genomics Superstruct Project (Buckner, 2012) demonstrated that efforts of similar or even grander scale can be undertaken while containing expenses. Specifically, the Superstruct effort added an optimized 15-min imaging acquisition protocol to ongoing studies at multiple sites and rapidly generated thousands of imaging datasets (Yeo et al., 2011). Web-based questionnaire and performance protocols were included to obtain comprehensive phenotyping while minimizing costs. The Mind Research Network (MRN; The Mind Research Network for Neurodiagnostic Discovery, 2012) provided another cost-effective model for large-scale data-generation by forming a collaborative (i.e., collaboration of laboratories) of independent investigators within and across multiple institutions united through the usage of a common informatics platform. Within the MRN, investigators can opt to share data with specific members, the larger collaborative or more broadly (e.g., see Allen et al., 2011). Finally, in recent years, uncoordinated, multi-center aggregation efforts such as the 1000 Functional Connectomes Project and its International Neuroimaging Data-sharing Initiative have emerged as open science solutions to the challenge of large-scale data aggregations (1000 Functional Connectomes Project (FCP), 2009; Biswal et al., 2010; Dolgin, 2010; Milham, 2012).

It is against this background that the NKI-RS emerged with the goal of building an institution-based open sharing model. The NKI-RS effort was designed to pool the global resources of an institution for the purpose of generating a large-scale, deeply phenotyped dataset reflective of the interests of its many investigators. Simultaneously, the open sharing of datasets was intended to facilitate investigations around the world and promote the generation and sharing of large-scale datasets at institution levels. The notion is that overlap of phenotypic protocols among openly shared datasets could rapidly accelerate the pace of discovery.

## NKI-Rockland Sample

In conceptualizing the NKI-RS initiative, a major focus was the creation of an institution-wide resource, reflective of the diverse interests of the NKI faculty, spanning pediatric, adult, and geriatric psychiatric illnesses. One key purpose was to create a data repository that could be used to test existing hypotheses as well as for generating novel hypotheses to spark new endeavors. The initial (pilot) phase of the NKI-RS was designed to demonstrate the feasibility of an institutionally based, discovery science project. Institutional support and resources were central to the success of the pilot, as it was conducted without dedicated external funding. The success of the pilot phase was instrumental in the attainment of NIMH funding for phase two, which embodies more sophisticated recruitment and sampling strategies, phenotyping, imaging, and neuroinformatics. The following section details the strengths and limitations of the first phase of the NKI-RS, as well as the design choices for the second phase.

## The NKI-RS Pilot (Phase I)

The goal of the pilot phase was to obtain diagnostic and behavioral assessments, tissue for genetic studies, and brain imaging (i.e., structural MRI, R-fMRI, diffusion imaging, and morphometry) on 250 individuals aged 4–89 years. More than 300 phenotypic variables were obtained across 26 psychiatric, behavioral, and cognitive domains. Additionally, participants consented in writing to unrestricted distribution of anonymous data through the International Neuroimaging Data-Sharing Initiative (INDI^1^; 1000 Functional Connectomes Project (FCP), 2009). Prospective data sharing for the pilot phase of the project began on a regular basis in October of 2010. In 11 months, the NKI-RS collected and released data from 250 individuals, demonstrating that the pace (approximately five datasets released per week) and the process of open pre-publication data sharing were feasible.

Despite its successes, the pilot phase of the NKI-RS also highlighted areas with substantial room for enhancement and innovation. First, phase one relied on a convenience sample consisting of any individual who was willing to participate within the designated age range (ages 4–89). This approach is vulnerable to recruitment biases that can diminish the representativeness of the acquired sample (Szklo, 1998; Evans and Brain Development Cooperative Group, 2006). Such biases can compromise the generalizability and reproducibility of findings. Efforts such as the NIH Normal Brain Development Study have demonstrated the feasibility and value of increasing representativeness through tracking and balancing regional demographics for participants based on zip code (Waber et al., 2007). Second, the phenotypic battery consisted primarily of convenience assessments based on current practices and availability, as well as the interests of individual NKI-RS investigators. In retrospect, it would have been preferable to use commonly available, normed, and validated assessments to increase their utility and overlap with those employed by other efforts in the research community. Third, the pilot phase used paper and pencil assessments, which required scoring, entering, and checking data by hand, a time-consuming and error-prone task. Further, the data entered into the database was limited to summary scores. The following sections discuss steps taken in the construction of the enhanced NKI-RS to address these limitations.

## The Enhanced NKI-RS (Phase II): Sampling and Recruitment

Phase two began in March 2012, with an anticipated 4-year project period to recruit 1000 participants. It was designed to yield a community-ascertained, lifespan sample (0.32% of the population) in which age, ethnicity, and socioeconomic status are representative of Rockland County, New York. Rockland County is a suburban/rural county 20 miles northwest of New York City, with a population of 311,687 per the 2010 Census. Fortuitously, ethnic and economic demographics of Rockland County resemble those of the United States (U.S. Census Bureau, 2010), increasing the generalizability of the NKI-RS to the broader U.S. population (Table 1).

**Table 1:** **2010 United States census data: Rockland County versus United States**.

People facts (Census, 2010)	Rockland county	USA
Population	311,687	308,745,538
Persons under 5 years old	7.6%	6.5%
Persons under 18 years old	28.1%	24.0%
Persons 65 years old and over	13.4%	13.0%
Female persons, percent, 2010	51.0%	50.8%
White	73.2%	72.4%
Black or African American	11.9%	12.6%
American Indian/Alaska Native	0.3%	0.9%
Asian	6.2%	4.8%
Native Hawaiian or other Pacific Islander	0.0%	0.2%
Two or more races reported	2.5%	2.9%
Hispanic or Latino	15.7%	16.3%
White, non-Hispanic	65.3%	63.7%
Foreign born, 2006–2010	22.1%	12.7%
Language other than English spoken at home	35.6%	20.1%
High school graduates	87.9%	85.0%
Bachelor’s degree or higher	40.7%	27.9%
Persons per household, 2006–2010	3.02	2.59
Median household income, 2006–2010	$82,534	$51,914
Per capita money income, 2006–2010	$34,304	$27,334
Persons below poverty level	11.3%	13.8%

*This figure contains 2010 census data for Rockland County in the State of New York as well as for the United States of America (USA). The purpose of this figure is to demonstrate that the census composition of Rockland County is similar to that of the USA as a whole. Therefore, data from this discovery science project based in Rockland County is likely to generalize to the USA*.

Following the model of efforts such as the NIH Normal Brain Development Study, zip code based recruitment (e.g., advertisement flyer mailings, posting of materials in local shops and meeting places) and enrollment efforts are being used to avoid over-representation of any portion of the community, and to ensure faithful representation of Rockland County. We monitor and adjust enrollment as necessary to ensure that the relative proportions of age, sex, and ethnicity accrued remain stable throughout the 4-years of this project, thereby minimizing potential cohort biasing effects (e.g., enrolling from one sub-population primarily in year one, and from another in year four).

Practical and logistical limitations dictated some constraints on the age range of our sample. Although children as young as 4 years of age were imaged successfully in the initial NKI-RS, we selected 6 years of age as the lower age limit to balance data losses with scientific yield. Similarly, we opted to truncate the upper limit at age 85 (versus 89) because of the dramatically increased rate of chronic illness above age 85. Additionally, the second phase of this project has intentional oversampling of the extremes of the lifespan (youngest and oldest) to increase statistical power for ages characterized by greatest changes (Table 2).

**Table 2:** **Enrollment strategy for enhanced NKI-RS**.

Age range	Target enrollment
6–10	150
11–20	150
21–30	100
31–40	75
41–50	100
51–60	125
61–70	150
71–85	150

*This figure shows the age range in years with corresponding enrollment targets for the NKI-RS. The total enrollment is 1000 individuals ages 6–85 years in a 4-year period. There is intentional oversampling at the ends of the age range due to the rapidity of developmental changes associated with youth and older age*.

## The Enhanced NKI-RS: Assessment

**Figure 1:**
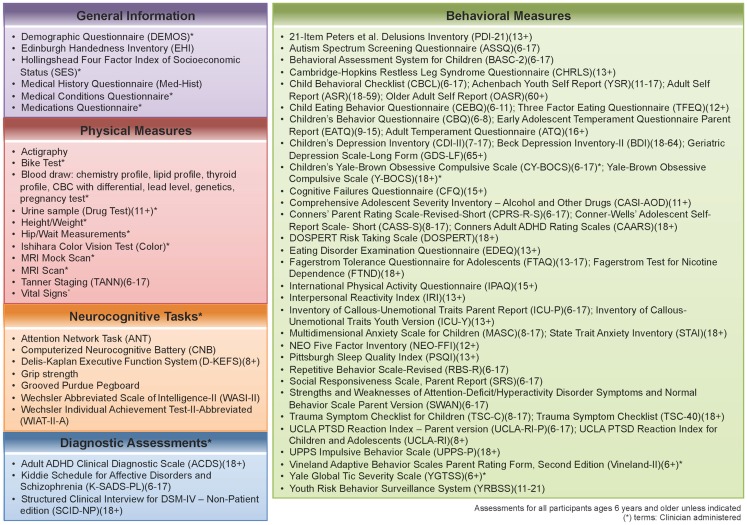
**Assessment protocol for NKI-RS**. This figure illustrates all of the assessments that are included in the 2-day enhanced Nathan Kline Institute-Rockland Sample (NKI-RS) protocol. There are five broad domains of assessment: General, Physical, Neurocognitive, Diagnostic, and Behavioral. Within the table are the names, abbreviations, and age ranges in years for each of the assessments.

Phase two of the NKI-RS project will contain broader and deeper phenotypic characterization of participants, with a focus on key psychiatric and neurocognitive constructs. The battery for the second phase was constructed based on discussions with assessment developers, expert consultants, and a formal presentation to the Child Mind Institute’s Scientific Research Council (SRC)^2^. We prioritized inclusion of empirically validated measures in the public domain, as those are most amenable to widespread adoption in other studies. Additionally, we prioritized use of measures that could be administered and compared across the lifespan. In attempting to serve as a resource for future studies, the second phase compares commonly used assessments that measure the same construct, behavior, or disorder. We also compare proprietary and non-proprietary assessments (e.g., the Conners ADHD Scales (Conners et al., 1997; Conners, 1999) versus Strengths and Weaknesses of ADHD-Symptoms and Normal-Behavior Scale (SWAN; Hay et al., 2007, respectively), and can assess the construct validity of different assessments (e.g., the Computerized Neurocognitive Battery (Gur et al., 2001) versus the Delis–Kaplan Executive Functioning System (Delis et al., 2004; Figure 1).

**Figure 2:**
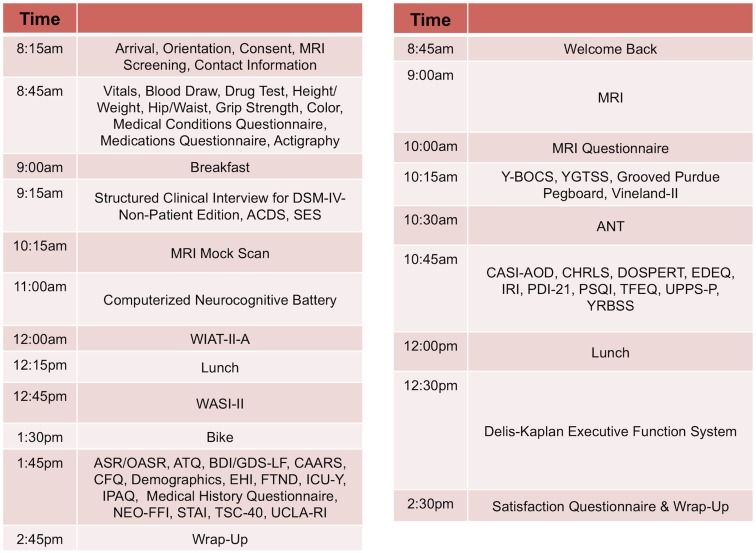
**Sample schedule for adult participants in NKI-RS**. This figure illustrates the 2-day assessment schedule for adult participants (ages 18–85 years) in the Nathan Kline Institute-Rockland Sample (NKI-RS) protocol. Abbreviations for the assessments: ANT, Attention Network Task; ASR, Adult Self Report; ATQ, Adult Temperament Questionnaire; BDI, Beck Depression Inventory-II; CAARS, Conners’ Adult ADHD Rating Scales; CASI-AOD, Comprehensive Adolescent Severity Inventory – Alcohol and Other Drugs; CHRLS, Cambridge-Hopkins Restless Leg Syndrome Questionnaire; CFQ, Cognitive Failures Questionnaire; DOSPERT, DOSPERT Risk Taking Scale; GDS-LF, Geriatric Depression Scale-Long Form; OASR, Older Adult Self Report; EDEQ, Eating Disorder Examination Questionnaire; EHI, Edinburgh Handedness Inventory; FTND, Fagerstrom Test for Nicotine Dependence; ICU-Y, Inventory of Callous-Unemotional Traits Youth Version; IPAQ, International Physical Activity Questionnaire; IRI, Interpersonal Reactivity Index; NEO-FFI, NEO Five Factor Inventory; PDI-21, 21-Item Peters et al. Delusions Inventory; PSQI, Pittsburgh Sleep Quality Index; STAI, State Trait Anxiety Inventory; TFEQ, Three Factor Eating Questionnaire; TSC-40, Trauma Symptom Checklist; UCLA-RI, UCLA PTSD Reaction Index for Children and Adolescents; UPPS-P, UPPS Impulsive Behavior Scale; Vineland-II, Vineland Adaptive Behavior Scales Parent Rating Form, Second Edition; WASI-II, Wechsler Abbreviated Scale of Intelligence-II; WIAT-II-A, Wechsler Individual Achievement Test-II-Abbreviated; Y-BOCS, Yale-Brown Obsessive Compulsive Scale; YGTSS, Yale Global Tic Severity Scale; YRBSS, Youth Risk Behavior Surveillance System.

**Figure 3:**
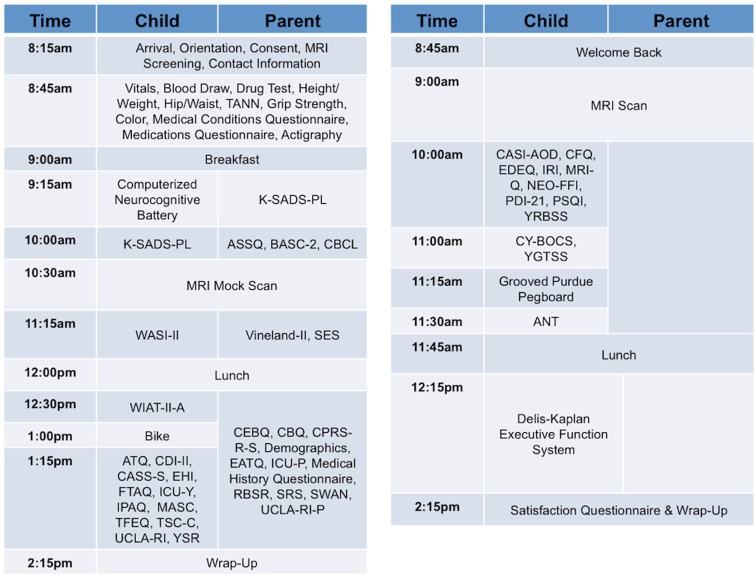
**Sample schedule for child and parent participants in NKI-RS**. This figure illustrates the 2-day assessment schedule for child and parent participants (children ages 6–17 years) in the Nathan Kline Institute-Rockland Sample (NKI-RS) protocol. Abbreviations for the assessments: ANT, Attention Network Task; ASSQ, Autism Spectrum Screening Questionnaire; ATQ, Adult Temperament Questionnaire; BASC-2, Behavioral Assessment System for Children; CASI-AOD, Comprehensive Adolescent Severity Inventory-Alcohol and Other Drugs; CASS-S, Conner-Wells’ Adolescent Self-Report Scale-Short; CBCL, Child Behavioral Checklist; CBQ, Children’s Behavior Questionnaire; CFQ, Cognitive Failures Questionnaire; CDI-II, Children’s Depression Inventory-II; CEBQ, Child Eating Behavior Questionnaire; CPRS-R-S, Conners’ Parent Rating Scale-Revised-Short; CY-BOCS, Children’s Yale-Brown Obsessive Compulsive Scale; EATQ, Early Adolescent Temperament Questionnaire Parent Report; EDEQ, Eating Disorder Examination Questionnaire; EHI, Edinburgh Handedness Inventory; FTAQ, Fagerstrom Tolerance Questionnaire for Adolescents; ICU-P, Inventory of Callous-Unemotional Traits Parent Report; ICU-Y, Inventory of Callous-Unemotional Traits Youth Version; IPAQ, International Physical Activity Questionnaire; IRI, Interpersonal Reactivity Index; K-SADS-PL, Kiddie Schedule for Affective Disorders and Schizophrenia; MASC, Multidimensional Anxiety Scale for Children; MRI-Q, Magnetic Resonance Imaging Questionnaire; NEO-FFI, NEO Five Factor Inventory; PSQI, Pittsburgh Sleep Quality Index; RBSR, Repetitive Behavior Scale-Revised; SES, Hollingshead Four Factor Index of Socioeconomic Status; SRS, Social Responsiveness Scale-Parent Report; SWAN, Strengths and Weaknesses of Attention-Deficit/Hyperactivity Disorder Symptoms and Normal-Behavior Scale-Parent Version; TANN, Tanner Staging; TFEQ, Three Factor Eating Questionnaire; TSC-C, Trauma Symptom Checklist for Children; UCLA-RI, UCLA PTSD Reaction Index for Children and Adolescents; UCLA-RI-P, UCLA PTSD Reaction Index–Parent version; Vineland-II, Vineland Adaptive Behavior Scales Parent Rating Form, Second Edition; WASI-II, Wechsler Abbreviated Scale of Intelligence-II; WIAT-II-A, Wechsler Individual Achievement Test-II-Abbreviated; YGTSS, Yale Global Tic Severity Scale; YRBSS, Youth Risk Behavior Surveillance System; YSR, Achenbach Youth Self Report.

Our approach, similar to that of the Brain Genomics Superstruct (Yeo et al., 2011), differs from the more common model of centering collaborative efforts on a particular disorder or set of disorders, which can limit the applicability of a comparison sample. Given the current focus on developing a dimensional framework for psychiatric illnesses and patterns of comorbidity (Chabernaud et al., 2012), we adopted broad phenotypic characterization for phase two of NKI-RS. By employing a common protocol that covers a wide array of domains of psychiatric, cognitive, and behavioral functions, we can make direct comparisons between psychiatric illnesses and increase the feasibility of determining overlap and distinctions among their neural correlates. During review of the finalized phenotyping protocol by the SRC, a key concern that emerged was that the comprehensiveness of the phenotyping protocol increased the burden to participants and experimenters – potentially endangering its effectiveness due to factors such as fatigue and increased data management needs. To address these concerns, the NKI-RS protocol was decompressed from a 1- to a 2-day format. As discussed below, state-of-the-art computer based data entry, scoring, and management capabilities were added, thereby minimizing burden on both participants and experimenters^3^. Additionally, we carried out focus-group testing prior to initiation of the sample, and are obtaining customer satisfaction surveys and monitoring participant feedback as we progress so that small tweaks to the protocol can be made as necessary (preferably within the first 100 participants; Figures 2 and 3).

A key innovation of the second phase NKI-RS is the implementation of fast repetition time (0.645 and 1.4 s TR) and high-resolution (3 and 2 mm isotropic voxels) multiband R-fMRI (10 min per scan), and DTI (137-direction, 2 mm isotropic) measures provided by the Center for Magnetic Resonance Research at the University of Minnesota for the Human Connectomes Project (Feinberg et al., 2010; Smith et al., 2012; see^4^). Additionally, NKI-RS included a brief visual-checkerboard stimulation scan (duration = 2 min) for each of the multiband sequences to allow for assessment of the contrast to noise ratio. The NKI-RS and HCP efforts are independent projects, the former primarily focused on examination of brain-behavior relationships across the lifespan, and the latter focused on a twin and family based study of genetic-brain-behavior relationships in young adults (Van Essen et al., 2012). Despite these differences in focus, it is anticipated that inclusion of fast TR protocols will increase the ability of scientists to maximize the areas of overlap and compare or aggregate data obtained from the HCP and NKI-RS samples.

## The Enhanced NKI-RS: Database and Data Sharing

The broad and deep phenotyping of the enhanced NKI-RS raised several issues regarding data entry and administration. First, we could not continue paper and pencil approaches with research assistants entering summary scores into spreadsheets, as this inevitably leads to errors, which are expensive to find, correct, or prevent. Additionally, the practice of logging summary scores alone is inherently flawed, as potentially valuable item-level information is lost and typically too expensive to recover later. Second, integrating phenotypic and imaging data is non-trivial, and an undesirable potential source of error for investigators (Marcus et al., 2007). Fortunately, packages such as XNAT (Marcus et al., 2007), LORIS (Longitudinal Online Research and Imaging System, 2011), HID (Helmer et al., 2011), and COINS (Scott et al., 2011) have emerged as viable options, though their usage is still relatively limited.

From these options, the NKI-RS team selected the Collaborative Informatics and Neuroimaging Suite (COINS) developed by the Mind Research Network (Scott et al., 2011; The Mind Research Network for Neurodiagnostic Discovery, 2012). COINS was created to facilitate communication and cultivate a data-sharing community by providing researchers with an open source information system that includes web-based tools to manage studies, subjects, imaging, and phenotypic data. This suite of tools has an intuitive ease of use and offers versatile data upload/import/entry options, rapid and secure sharing of data among investigators, querying of data types and assessments, real-time reporting, and study-management tools. Among its many features, the web-based assessments, automated data scoring, and integrated management of phenotypic and imaging data are potentially the most attractive. Web-based assessment entry completed by participants and research staff increases efficiency and accuracy by eliminating the need for intermediate data entry (i.e., paper to computer). Equally important, individual item-level responses are coded in the database, providing researchers with a far richer phenotypic dataset for exploration. In addition, protected health information can be unlinked within COINS to facilitate data sharing while maximally protecting participant anonymity. Of note, COINS is in compliance with Health Insurance Portability and Accountability Act (HIPAA) standards and implementation rules.

Confidentiality was a paramount consideration in planning data-sharing requirements for phase two. Protecting participant privacy while also providing access to extensively revealing data was a goal. In the pilot phase, all imaging data were fully anonymized in compliance with HIPAA by removing any potential protected health information identifiers, including identifying facial features from anatomical images, and randomizing the timing of release. It is important to note that data users must be aware of the possible negative impact of defacing on some analysis toolkits (e.g., FreeSurfer), and exercise additional care when producing such images and/or sharing pre-processed surfaces. Summary scores from all such measures were made publicly available, along with individuals’ imaging data^5^. The same anonymization and distribution protocol was used in December of 2012, when the Enhanced NKI-Rockland team released a 24-participant multiband imaging test-retest pilot dataset, created to evaluate the cutting edge “fast TR” sequences provided by the Human Connectome Project for usage in the enhanced sample^6^.

Although successful for the pilot efforts, the phase two NKI-RS effort has two unique features that called for reconsideration of the data-sharing policy. First, the Enhanced NKI-RS differs from the pilot sample in that it is being obtained using a community-ascertained epidemiologic design, which requires residence in Rockland County. Accordingly, a given participant’s residential location is identifiable to the level of a county, which is not in keeping with the definition of complete de-identification based on HIPAA’s 18 protected health identifiers. Second, the concurrently supplied psychometric data in the Enhanced NKI-RS phenotypic protocol will include individual item-level data and an increased breadth of phenotypic sampling relative to the pilot NKI-RS efforts. The high-dimensionality of these data increases risk of identification far beyond that posed by revealing some of the 18 protected health identifiers specified by HIPAA. These concerns support the need for implementation of a data use agreement.

Given these considerations, the Enhanced NKI-RS Sample is requiring a data usage agreement for access to the data – a requirement similar to efforts such as the Alzheimer’s Disease Neuroimaging Initiative (ADNI) and the National Database for Autism Research (NDAR) 2009,^7^. The adoption of a data usage agreement is not intended to limit the specific analyses a researcher can perform; users will only need to specify the broad range of analyses they may pursue with the data (e.g., association studies between DTI, R-fMRI, and behavior), not a specific analysis or set of analyses. The intent of the agreement is to ensure that data users agree to protect participant confidentiality when handling data that contains potentially identifying information and that they will agree to take the necessary measures to prevent breaches of privacy. The specific agreement to be employed for the Enhanced NKI-RS are those previously defined by the New York State Office of Mental Health, which consist of two straightforward components: A Data Exchange Agreement and a Non-Disclosure of Confidential Information Agreement (forms can be found at^8^^,^^9^). Unlike the NDAR agreement, institutional review board (IRB) approval is not required for transfer of the data; it will be up to the individual data user to satisfy any additional requirements specified by their local IRB or ethics committee, prior to using the NKI-RS. Given that local IRB approval is not required as part of an individuals application for access to the NKI-RS, there is no need for an individual’s IRB to have a federal-wise assurance number – which can limit recipients of the NDAR datasets.

## Ethics Statement

Institutional Review Board Approval was obtained for this project at the Nathan Kline Institute (Phase I #226781 and Phase II #239708) and at Montclair State University (Phase I #000983A and Phase II #000983B). Written informed consent was obtained for all study participants. Written consent and assent was also obtained from minor/child participants and their legal guardian.

## A Note on Error-Handling

Fears and attitudes regarding the reporting of data errors represent a major obstacle to open data sharing (Poldrack, 2011). Despite any group’s best efforts to prevent errors from arising in the process of acquiring, handling, and distributing data, errors will undoubtedly arise. The pilot phase of the NKI-RS had to face such a challenge, when a slight deviation in the scoring of the DKEFS was noted. Although this error was relatively inconsequential, the team worked to rapidly report^10^ and correct it. Indeed, even relatively small errors in publicly shared data must be reported as soon as they are discovered, so that the community can be confident that the accuracy of the shared data is the best attainable. Without monitoring and ongoing open error reporting, errors will be perpetuated and their impact potentially magnified (Friedman and Glover, 2006).

## Conclusion

Although the generation and release of data for 1000 broadly and deeply phenotyped participants with extensive neuroimaging data and archived genetic samples requires substantial effort, it is just the beginning of one project among the many needed to fully unravel the neurobiology of psychiatric disorders across the lifespan. The NKI-RS is intended to serve as a jumping off point for research that goes beyond individual institutions and has the power to obtain the truly large numbers needed to create normative trajectories in psychiatry. Attainment of normative lifespan trajectories will have a transformational effect on the way in which neuropsychiatric research is conducted (Gogtay et al., 2004; Sowell et al., 2004; Evans and Brain Development Cooperative Group, 2006; Shaw et al., 2008, 2010; Biswal et al., 2010; Giedd and Rapoport, 2010).

Projects like the NKI-RS have the power to inform our understanding of the entire spectrum of psychiatric illness. It is our hope that the NKI-RS framework will inspire other institutions to join the era of discovery and revolutionize clinical practice for all of psychiatry, from children and adolescents to older adults. The ability to relate dimensional phenotypic measures to statistically normed brain relationships will support the identification of underlying pathophysiological mechanisms, which may ultimately transform psychiatric nosology, guide the diagnostic process, inform treatment selection, and permit tracking of therapeutic efficacy.

## Conflict of Interest Statement

The authors declare that the research was conducted in the absence of any commercial or financial relationships that could be construed as a potential conflict of interest.

## Footnotes

^1^http://fcon_1000.projects.nitrc.org

^2^http://www.childmind.org/en/directory/src/

^3^Measures taken to minimize subject and experimenter burden in response to CMI SRC recommendations (e.g., decompression from 1- to 2-day protocol, enhancement of informatics) were made possible by a grant provided by the Child Mind Institute, Inc (1FDN2012-1).

^4^http://fcon_1000.projects.nitrc.org/indi/pro/eNKI_RS_TRT/FrontPage.html for protocol specifications

^5^http://fcon_1000.projects.nitrc.org/indi/pro/nki.html

^6^http://fcon_1000.projects.nitrc.org/indi/pro/eNKI_RS_TRT/FrontPage.html

^7^http://ndar.nih.gov/

^8^http://rocklandsample.rfmh.org/RocklandSample_dea.pdf

^9^http://rocklandsample.rfmh.org/RocklandSample_cnda.pdf

^10^http://www.nitrc.org/forum/forum.php?thread_id=2600&forum_id=1735
